# Effect of pirfenidone on lung function decline and survival: 5-yr experience from a real-life IPF cohort from the Czech EMPIRE registry

**DOI:** 10.1186/s12931-019-0977-2

**Published:** 2019-01-21

**Authors:** Monika Zurkova, Eva Kriegova, Vitezslav Kolek, Vladimira Lostakova, Martina Sterclova, Vladimir Bartos, Martina Doubkova, Ilona Binkova, Michal Svoboda, Jana Strenkova, Marketa Janotova, Martina Plackova, Ladislav Lacina, Vladimir Rihak, Frantisek Petrik, Pavlina Lisa, Radka Bittenglova, Richard Tyl, Gustav Ondrejka, Hana Suldova, Jaroslav Lnenicka, Jana Psikalova, Tomas Snizek, Jiri Homolka, Renata Kralova, Jan Kervitzer, Martina Vasakova, ILD section, IPF registry

**Affiliations:** 1 Department of Pulmonary Diseases and Tuberculosis, Faculty of Medicine and Dentistry, Palacky University in Olomouc, University Hospital Olomouc, Olomouc, Czech Republic; 2 Department of Immunology, Faculty of Medicine and Dentistry, University Hospital Olomouc, Palacky University in Olomouc, Hnevotinska 3, 775 15 Olomouc, Czech Republic; 3 Department of Respiratory Medicine, 1st Faculty of Medicine Charles University in Prague, Thomayer Hospital, Prague, Czech Republic; 4 Department of Pulmonary Medicine, Faculty of Medicine in Hradec Kralove at Charles University in Prague, University Hospital Hradec Kralove, Prague, Czech Republic; 5 Department of Pulmonary Diseases and Tuberculosis, Faculty of Medicine at Masaryk University, University Hospital Brno, Brno, Czech Republic; 6 Institute of Biostatistics and Analyses, Faculty of Medicine, Masaryk University, Brno, Czech Republic; 7 Department of Pulmonary Diseases and Tuberculosis, Faculty of Medicine at University of Ostrava, University Hospital Ostrava, Ostrava, Czech Republic; 8 Department of Pulmonary Medicine and Thoracic Surgery, Hospital Na Bulovce, Prague, Czech Republic; 9 Department of Pulmonary Medicine, Tomas Bata Regional Hospital, Zlin, Czech Republic; 10 Department of Pulmonary Medicine, 2nd Faculty of Medicine at Charles University in Prague, University Hospital in Motol, Prague, Czech Republic; 11 Department of Pulmonary Medicine, University Hospital Plzen, Pilsen, Czech Republic; 12 Department of Pulmonary Medicine, Hospital Novy Jicin, Novy Jicin, Czech Republic; 13 Department of Pulmonary Medicine, Hospital Ceske Budejovice, Ceske Budejovice, Czech Republic; 14 Department of Pulmonary Diseases and Tuberculosis, Regional Medical Association, JSC – Masaryk Hospital in Usti nad Labem, Usti nad Labem, Czech Republic; 15 Department of Pulmonary Medicine and Allergology, Hospital Kromeriz, Kromeriz, Czech Republic; 16 Department of Pulmonary Medicine, Hospital Jihlava, Jihlava, Czech Republic; 17 Department of Pulmonary Medicine, Regional Hospital Pardubice, Pardubice, Czech Republic; 18 Department of Pulmonary Medicine, Hospital Znojmo, Znojmo, Czech Republic

**Keywords:** Idiopathic pulmonary fibrosis, Pirfenidone, Mortality prediction, Disease progression

## Abstract

**Introduction:**

Pirfenidone, an antifibrotic drug, slows-down the disease progression in idiopathic pulmonary fibrosis (IPF) over 12 months, however limited data on the decline of lung function and overall survival (OS) in real-world cohorts on longer follow-up exists.

**Patients/methods:**

Of the enrolled Czech IPF patients (*n* = 841) from an EMPIRE registry, 383 (45.5%) received pirfenidone, 218 (25.9%) no-antifibrotic treatment and 240 (28.5%) were excluded (missing data, nintedanib treatment). The 2- and 5-yrs OS and forced vital capacity (FVC) and diffusing lung capacity for carbon monoxide (DL_CO_) were investigated at treatment initiation and 6, 12, 18 and 24 months’ follow-up.

**Results:**

During a 2-yr follow-up, less than a quarter of the patients progressed on pirfenidone as assessed by the decline of ≥10% FVC (17.0%) and ≥ 15% DL_CO_ (14.3%). On pirfenidone, the DL_CO_ (≥10%) declines at 6, 12, 18 and 24 months’ and DL_CO_ (≥15%) declines at 6, 18 and 24 months’ follow-up were associated with increased mortality. The DL_CO_ decline showed higher predictive value for mortality than FVC decline. In patients with no-antifibrotics, FVC and DL_CO_ declines were not predictive for mortality. Pirfenidone increased 5-yrs OS over no-antifibrotic treatment (55.9% vs 31.5% alive, *P* = 0.002).

**Conclusion:**

Our study observed the 2-yrs sustained effect of pirfenidone on the decline of lung function and survival in the real-world patient’s IPF cohort. DL_CO_ decline of ≥10% shows a potential as a mortality predictor in IPF patients on pirfenidone, and should be routinely evaluated during follow-up examinations.

## Introduction

Idiopathic pulmonary fibrosis (IPF) belongs to the idiopathic interstitial pneumonias; it is characterised by a progressively declining lung function, leading to respiratory failure and death, with a median survival of 2–3 years following initial diagnosis [1, 2]. Epidemiological data in many countries are likely underestimated as the diagnosis of IPF tends to be mistaken or missed completely [3] thus an extensive need to establish national and international registries occurred. The Czech IPF registry was launched in June 2012, and on its basis the EMPIRE (*European MultiPartner IPF Registry*) project was launched in 2014 aiming at the assessment of IPF incidence, prevalence, and mortality in Central and Eastern Europe, the determination of basic patients’ characteristics and used treatment (http://empire.registry.cz/index-en.php).

Since 2015, antifibrotic therapy with either pirfenidone or nintedanib has been recommended in IPF patients [2, 4]. Although the treatment with pirfenidone is not curative, the clinical trials showed that it reduces disease progression, as reflected by lung function, exercise tolerance, and progression-free survival [5–7] and improves life expectancy [8]. There is a growing body of evidence that early diagnosis and early initiation of treatment by pirfenidone may preserve lung functional status and prolong life. A positive effect of pirfenidone on disease progression was also recently observed in patients with more advanced disease with less preserved lung function [9–12].

The effectiveness of pirfenidone in terms of reduced forced vital capacity (FVC) decline, decline in 6-min’ walk test distance (6-MWT) and increase of progression-free survival at 12 months after administration has been proven in Phase III CAPACITY/ASCEND population [7, 8] and cohorts from Belgium and the Netherlands [13], Italy [11], Japan [14], Germany [15], the UK [16], Sweden [17], Denmark [18], and Greece [12]. However, long-term data on disease progression in IPF patients treated with pirfenidone is still limited [19, 20].

We therefore investigated 2- and 5-yrs overall survival and lung function declination in IPF patients during long-term treatment with i) pirfenidone and ii) no-antifibrotic drugs. Additionally, the relationship between lung function declination and mortality was estimated. Data was collected prospectively from IPF patients enrolled in the Czech IPF registry between December 2012 to December 2017 in a real-life approach.

## Patients and methods

**Fig. 1:**
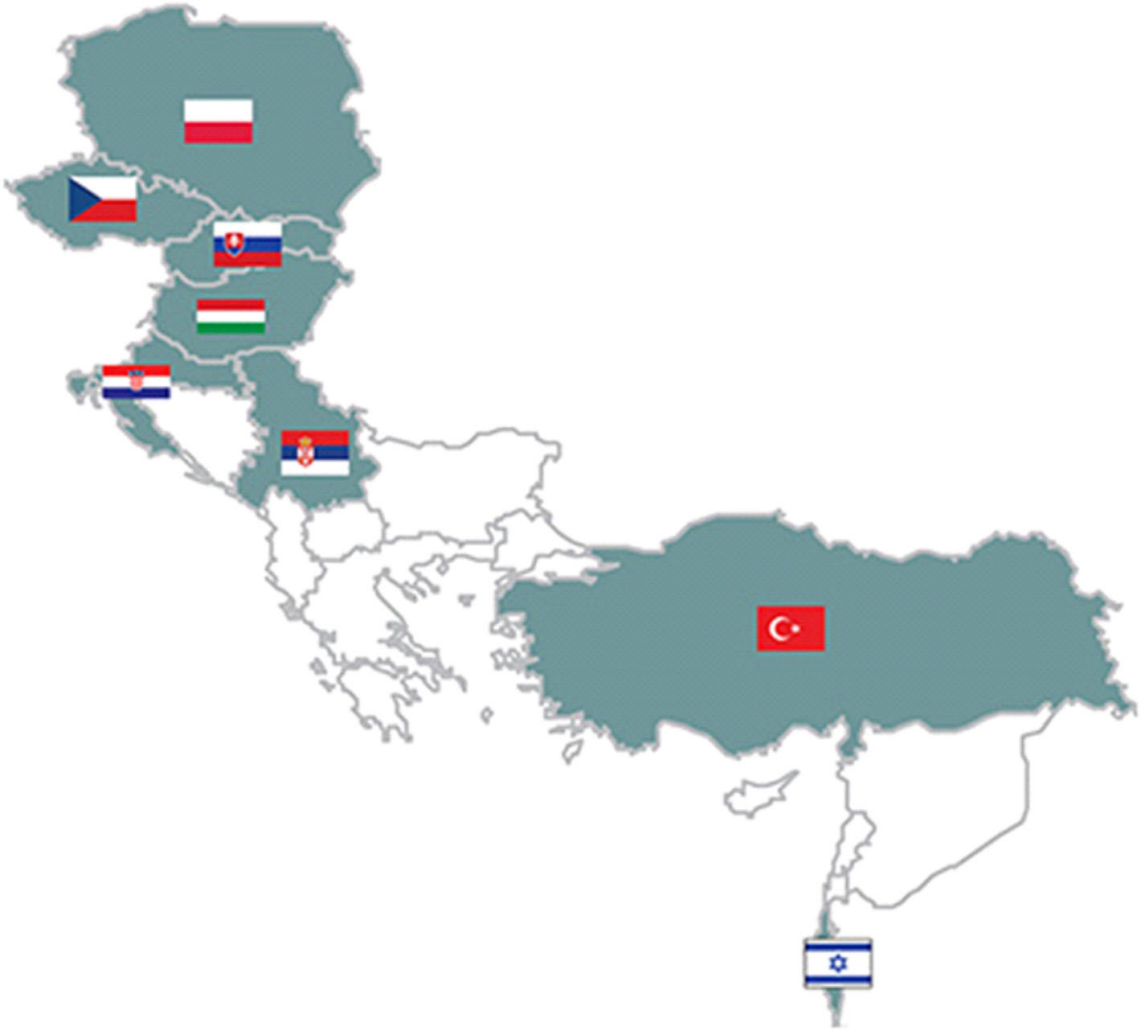
Participating countries in the **EMPIRE** project

In this study, IPF patients (*n* = 841) from the Czech IPF registry, a national registry within an international multicentre database of patients with IPF in Central and Eastern Europe **(**EMPIRE, *European MultiPartner IPF Registry)* were enrolled (Fig. 1). All patients were diagnosed according to the American Thoracic Society (ATS)/European Respiratory Society (ERS) consensus classification [1] in 15 centres for interstitial lung diseases (ILDs) in the Czech Republic between December 2012 to December 2017. The complete clinical and demographical data (age, gender, treatment start, type of treatment, lung function before and during treatment, comorbidities) were available in 601 patients. 240 patients (28.5%) were excluded from further analysis due to a change of diagnosis (*n* = 6), unknown type of treatment or unknown date of treatment start (*n* = 89) or nintedanib treatment (*n* = 145). Of those with complete clinical and demographical data, 383 (63.7%) patients were treated with pirfenidone and 218 (36.3%) patients treated with other modalities (corticosteroids, N-acethylcystein (NAC), azathioprine and their combinations). Patient demographic and clinical characteristics are shown in Table 1. The assessment of forced vital capacity (FVC) and diffusing lung capacity for carbon monoxide (DL_CO_) and mortality in enrolled patients were investigated at treatment initiation (max 1 month to treatment start) with 6 (±1), 12 (±1), 18 (±2), and 24 (±2) months follow-up. Disease progression as measured by FVC decline was considered at two values (≥5%, ≥10%) and/or by DL_CO_ decline at two values (≥10%, ≥15%) [21–23]. Patients on nintedanib were not included in this study because this drug was commercially available in the Czech Republic after September 2016, and most patients on this treatment were followed for less than one year at the end of this study.

**Table 1:** Demographic data on IPF patients evaluated in this study

Number of patients (%)	Total	Pirfenidone treatment	No-antifibrotic treatment	*P*-value (pirfenidone vs no-antifibrotics)
Patients, %	601 (100.0%)	383 (63.7%)	218 (36.3%)	
Gender (male/female), number (%)	431 (71.7%)/170 (28.3%)	281 (73.4%)/102 (26.6%)	150 (68.8%)/68 (31.2%)	0.258
Age (< 60/60–69/> 70 yrs)	106/236/259	66/157/160	40/79/99	0.520
Smoking status				0.057
Non smokers	275 (45.8%)	166 (43.3%)	109 (50.0%)	
Exsmokers	316 (52.6%)	213 (55.6%)	103 (47.2%)	
Smokers	10 (1.7%)	4 (1.0%)	6 (2.8%)	
FVC (%), predicted, at the treatment initiation Mean ± SD	71.7 ± 15.9	70.7 ± 13.2	74.2 ± 20.8	0.068
DL_CO_ (%), predicted, at the treatment initiation Mean ± SD	46.1 ± 13.6	47.8 ± 12.1	41.9 ± 16.1	< 0.001
FVC ≥50% and ≤ 90% predicted & DL_CO_ ≥ 35%, yes/no, number (%)	432/169 (71.9%/28.1%)	322/61 (84.1%/15.9%)	110/108 (50.5%/49.5%)	< 0.001

In July 2012, the first Czech patients with IPF were selected for pirfenidone therapy in the frame of The European Named Patient Programme (InterImmune International AG) and continued from July 2014 with a commercially available drug. The Czech guidelines for treatment of IPF from 2014 recommend pirfenidone treatment for patients with measured FVC ≥50% and ≤ 90% predicted and DLco ≥35% [24]. Pirfenidone was administrated at 2403 mg/day in 3 doses. The majority of patients enrolled by December 2014 obtained together with pirfenidone NAC at 3 × 600 mg based on recommendation from the clinical trials [25] and Czech IPF guidelines [24]. Since January 2015, pirfenidone has been administrated without NAC, according to the new evidence from clinical trials, mainly PANTHER trial [26, 27] and updated Czech IPF guidelines [28].

### Statistics

For a basic description of the input characteristics of patients, the differences in continuous variables were tested using the independent t-test/Mann-Whitney *U* test and the difference between two different time points by paired t-test/Wilcoxon paired test, based on the normality of the data assessed by the Shapiro–Wilk test. The differences in frequencies in categorical variables between groups were tested using Fisher’s exact test (four-field tables). Survival analysis was established using the Kaplan-Meier survival diagram, using the treatment initiation time as the starting point and the end of follow-up or the last clinical follow-up visit as the endpoints. Statistical significance tested by Log Rank test and the median survival time is presented. The analysis was prepared in IBM SPSS Statistics 24.0.0.0 with level of significance α = 0.05.

## Results

### Lung functions and its decline during treatment in IPF patients

Of the patients enrolled into the EMPIRE registry, the data on disease progression and other clinical data was available in 601 patients. The length of follow-up and the number of patients for whom data were available in the given follow-up months is shown in Table 2.

**Table 2:** Length of follow-up of enrolled patients

	Pirfenidone treatment	No-antifibrotic treatment
Length of follow-up (months, mean ± SD)	22.8 ± 16.5	32.1 ± 37.3
Number of patients at the treatment initiation	383 (100.0%)	218 (100.0%)
at posttreatment follow-up (in months):		
6 (±1) mo	331 (86.4%)	174 (79.8%)
12 (±1) mo	270 (70.5%)	144 (66.1%)
18 (±2) mo	204 (53.3%)	117 (53.7%)
24 (±2) mo	138 (36.0%)	98 (45.0%)
60 (±3) mo	17 (4.4%)	32 (14.7%)

### Lung function decline in the subgroups of IPF patients

In order to assess the lung function decline in patients on pirfenidone and those receiving no-antifibrotic treatment during the follow-up, the differences between FVC and DL_CO_ values at the treatment initiation and at 6, 12, 18 and 24-month’s follow-up were evaluated.

On pirfenidone, the percentages of patients who experienced a ≥ 10% decline in FVC were at 6, 12, 18 and 24 months as follows: 5.3, 10.7, 16.6, 17.0%, respectively (Table 3). In those with no-antifibrotic treatment, ≥10% decline in FVC was observed at 6, 12, 18 and 24 months for 18.3, 25.0, 24.2, 31.0% patients. When evaluating ≥5% decline in FVC as cut-off for progression, the percentages of the patients with progression over time were at 6, 12, 18 and 24 months (17.3, 25.2, 35.5, 34.8%) comparing to no-antifibrotic treatment (35.0, 44.4, 51.5, 50.0%) (Table 3).

**Table 3:** The lung function decline and progression as assessed by decline of A) FVC %, predicted, and B) DL_CO_ %, predicted, in groups of patients on pirfenidone and no-antifibrotic treatments

		Pirfenidone treatment	No-antifibrotic treatment	*P*-value (pirfenidone vs no-antifibrotics)
A) FVC %, predicted				
6 mo	Decline FVC %, predicted	1.0 ± 8.2*	− 2.1 ± 10.2	0.028
	% patients with decline ≥5%/decline < 5%	17.3%/82.7%	35.0%/65.0%	0.004
	% patients with decline ≥10%/decline < 10%	5.3%/94.7%	18.3%/81.7%	0.558
12 mo	Decline FVC %, predicted	0.2 ± 9.2	− 4.9 ± 10.5*	< 0.001
	% patients with decline ≥5%/decline < 5%	25.2%/74.8%	44.4%/55.6%	0.003
	% patients with decline ≥10%/decline < 10%	10.7%/89.3%	25.0%/75.0%	0.064
18 mo	Decline FVC %, predicted	−1.1 ± 10.4	− 6.0 ± 11.1*	0.016
	% patients with decline ≥5%/decline < 5%	35.5%/64.5%	51.5%/48.5%	0.116
	% patients with decline ≥10%/decline < 10%	16.6%/83.4%	24.2%/75.8%	0.818
24 mo	Decline FVC %, predicted	−0.9 ± 10.7	−6.7 ± 11.3*	0.003
	% patients with decline ≥5%/decline < 5%	34.8%/65.2%	50.0%/50.0%	0.102
	% patients with decline ≥10%/decline < 10%	17.0%/83.0%	31.0%/69.0%	0.420
B) DL_CO_ (%), predicted				
6 mo	Decline DL_CO_ (%), predicted	−1.6 ± 10.0*	− 4.6 ± 10.3*	0.036
	% patients with decline ≥10%/decline < 10%	15.4%/84.6%	18.6%/81.4%	0.558
	% patients with decline ≥15%/decline < 15%	6.1%/93.9%	8.5%/91.5%	0.559
12 mo	Decline DL_CO_ (%), predicted	−2.4 ± 11.0*	− 6.0 ± 11.6*	0.021
	% patients with decline ≥10%/decline < 10%	19.7%/80.3%	30.8%/69.2%	0.064
	% patients with decline ≥15%/decline < 15%	11.3%/88.7%	18.5%/81.5%	0.143
18 mo	Decline DL_CO_ (%), predicted	−3.6 ± 12.1*	− 6.1 ± 12.6*	0.294
	% patients with decline ≥10%/decline < 10%	22.4%/77.6%	25.0%/75.0%	0.818
	% patients with decline ≥15%/decline < 15%	11.5%/88.5%	12.5%/87.5%	0.772
24 mo	Decline DL_CO_ (%), predicted	−4.7 ± 11.8*	− 8.5 ± 10.1*	0.072
	% patients with decline ≥10%/decline < 10%	26.3%/73.7%	33.3%/66.7%	0.420
	% patients with decline ≥15%/decline < 15%	14.3%/85.7%	25.6%/74.4%	0.142

*Indicates significant difference between the treatment initiation and particular posttreatment time point

Progression is defined as decline of i) ≥ 5% or 10% FVC %, predicted or ii) ≥ 10% or 15% DL_CO_ (%), predicted

When using the DL_CO_ decline ≥15% as the marker of progression, the percentage of patients on pirfenidone who progressed at 6, 12, 18 and 24 months were:6.1, 11.3, 11.5, 14.3%, respectively. Percentage of patients who progressed with ≥15% decline in DL_CO_ on no-antifibrotic therapy at 6, 12, 18 and 24 months were as follows: 8.5, 18.5, 12.5, 25.6%. Using the ≥10% decline in DL_CO_, the percentages of patients on pirfenidone who progressed were at 6, 12, 18 and 24 months: 15.4, 19.7, 22.4, 26.3%, respectively. The percentages of patients who progressed on no-antifibrotic therapy were at 6, 12, 18 and 24 months: (18.6, 30.8, 25.0, 33.3%) (Table 3).

### The association between FVC and DL_CO_ declines and mortality in IPF patients

Next, we investigated the decline of FVC and DL_CO_ in groups of patients treated with pirfenidone as well as those on no-antifibrotic treatment and its association with mortality at 6, 12, 18 and 24 months.

The mortality in patients on pirfenidone as well as on no-antifibrotic drugs was not associated with progression as assessed by the FVC decline of 5%, predicted at any of investigated follow-up time point during 2-yrs follow-up **(**Table 4A). Similarly, a FVC decline of 10%, predicted was not associated with mortality, except for 24 months’ follow-up only in pirfenidone group (Table 4A). Regarding DL_CO_, the mortality in pirfenidone patients was associated with the decline ≥10% at 6, 12, 18 and 24 months’ follow-up (Table 4B). Also decline ≥15% DL_CO_ was predictive for mortality on pirfenidone at 6, 18 and 24 months (*P* < 0.05), but it did not reach significance at 12 months’ follow-up (*P* = 0.182) (Table 4B). In the no-antifibrotic-treated group, the decline ≥10% and ≥ 15% DL_CO_ showed a high predictive value for mortality only at 6 months’ follow-up (Table 4B).

**Table 4:** Relationship between (A) FVC, % predicted and (B) DL_CO_, % predicted and mortality in enrolled IPF patients treated with pirfenidone and no-antifibrotic drugs

A)	Pirfenidone	*P*-value	No antifibrotic treatment	*P*-value
Mortality				
No	73.8 (52.1; 95.3)	<0.001	80.5 (45.7; 111.6)	0.050
Yes	63.0 (45.9; 81.2)		69.2 (40.6; 101.1)	
Decline of FVC ≥10%				
6mo				
No	71.6 (48.6; 91.6)	0.901	90.6 (53.6; 121.0)	0.057
Yes	67.5 (55.8; 89.1)		78.2 (45.7; 106.5)	
12mo				
No	74.7 (54.1; 95.4)	0.120	82.0 (56.9; 121.0)	0.237
Yes	71.6 (48.6; 95.3)		78.3 (45.1; 107.2)	
18mo				
No	72.8 (57.7; 88.2)	0.671	81.6 (57.5; 117.0)	0.501
Yes	71.6 (50.1; 97.3)		76.5 (46.5; 119.4)	
24mo				
No	78.1 (51.3; 95.7)	0.020	78.2 (46.5; 98.6)	0.596
Yes	71.4 (49.7; 92.5)		72.3 (45.1; 102.8)	
Decline of FVC ≥5%				
6mo				
No	72.1 (50.0; 93.4)	0.494	86.9 (53.6; 117.0)	0.170
Yes	71.4 (48.6; 88.8)		76.5 (41.2; 119.4)	
12mo				
No	71.3 (53.2; 97.3)	0.557	81.6 (56.9; 119.4)	0.282
Yes	72.2 (49.7; 92.3)		73.2 (43.3; 105.0)	
18mo				
No	72.0 (53.6; 98.4)	0.242	81.0 (46.5; 119.4)	0.773
Yes	71.6 (49.7; 95.3)		78.6 (41.2; 119.9)	
24mo				
No	75.2 (50.0; 95.7)	0.209	80.1 (53.6; 98.6)	0.213
Yes	72.0 (49.7; 92.5)		70.8 (45.1; 95.2)	
B)	Pirfenidone	*P*-value	No antifibrotic treatment	*P*-value
Mortality				
No	48.1 (33.9; 73.5)	<0.001	38.0 (22.1; 70.4)	0.485
Yes	40.9 (30.3; 52.1)		41.3 (17.8; 62.0)	
Decline of DL_CO_ ≥15%				
6mo				
No	55.3 (36.9; 82.6)	0.021	52.2 (47.7; 56.4)	0.264
Yes	45.9 (33.2; 71.0)		41.4 (20.1; 65.7)	
12mo				
No	49.0 (33.4; 81.0)	0.182	55.7 (29.5; 71.8)	0.108
Yes	46.0 (33.9; 71.1)		43.6 (18.9; 65.9)	
18mo				
No	59.7 (37.7; 82.6)	0.001	61.3 (47.4; 71.8)	0.046
Yes	46.4 (33.9; 72.5)		41.4 (15.6; 63.9)	
24mo				
No	61.4 (36.3; 83.7)	0.002	50.6 (35.4; 67.6)	0.247
Yes	47.0 (33.2; 69.1)		42.4 (21.4; 65.7)	
Decline of DL_CO_ ≥ 10%				
6mo				
No	56.3 (35.4; 81.0)	0.004	52.2 (39.2; 60.5)	0.077
Yes	45.8 (32.4; 67.3)		38.6 (20.1; 65.7)	
12m**o**				
No	51.4 (35.7; 83.7)	0.017	54.3 (30.2; 69.7)	0.147
Yes	45.8 (33.5; 66.8)		40.4 (18.9; 65.9)	
18mo				
No	56.0 (34.5; 81.0)	0.041	51.8 (29.6; 71.8)	0.223
Yes	46.5 (33.9; 72.8)		41.4 (15.6; 63.9)	
24mo				
No	55.2 (36.3; 83.7)	0.012	48.6 (35.4; 67.6)	0.108
Yes	46.9 (32.4; 67.3)		40.1 (21.4; 63.9)	

Differences between two groups were tested by Mann-Whitney test

### Number of hospitalizations and their reasons in treated IPF patients

Next, we evaluated the number of hospitalized patients and hospitalisation rates in groups of patients on pirfenidone and those with no-antifibrotic drugs, with special emphasis to acute exacerbations.

The comparison of percentages of hospitalized patients treated with pirfenidone (patients/percentage from whole subgroup: 122/31.9%) and those on no-antifibrotic treatment (86/39.4%) showed trend to lower percentages of hospitalized patients in pirfenidone group (*P* = 0.062). The percentage of patients hospitalized for acute exacerbations did not reach significance between pirfenidone (70/18.3%) and no-antifibrotic groups (53/24.3%, *P* = 0.092). Regarding the number of exacerbations, less patients on pirfenidone had one exacerbation (41/10.7% vs 30/13.8%), 2 exacerbations (21/5.5% vs 14/6.4%), and ≥ 3 exacerbations (8/2.1% vs 9/4.2%) then patients on no-antifibrotics, but the differences did not reach significance (*P* = 0.371).

### Overall survival and reason for end of follow-up in enrolled patients

Next, we analysed the overall survival of patients on pirfenidone and those receiving no-antifibrotic treatments as well as investigated the reason for end-of-follow-up in both groups.

**Fig. 2:**
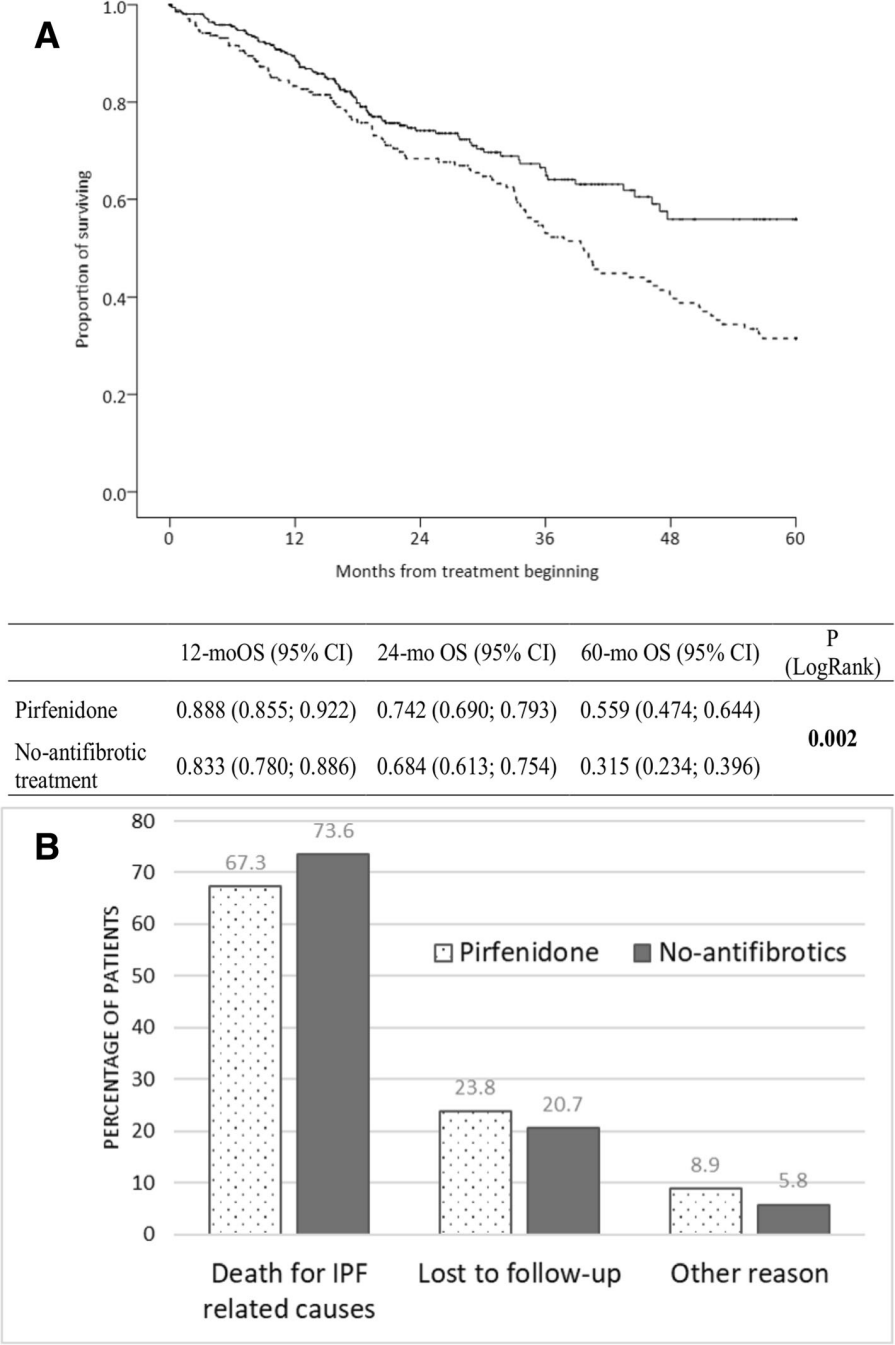
**a**) Overall survival of IPF patients on pirfenidone (full line) and no-antifibrotic treatment (dashed line), **b**) Reason for end of follow-up of enrolled IPF patients

The pirfenidone treated patients had longer overall survival, evident at 12, 24 and 60 months after treatment initiation, comparing to those treated with no-antifibrotic drugs (*P* = 0.002) (Fig. 2a). After 5-yrs, the median survival was not reached in the pirfenidone group with more than half of patients still alive after 5-yrs (55.9%) vs one-third on no antifibrotic treatment (31.5%) (Fig. 2a). End of follow-up was recorded in 141 (36.8%) of patients treated with pirfenidone and 161 (73.9%) of those with no-antifibrotic treatment (Table 5). In both groups, the main reason for end of follow-up was death (26.4% in the pirfenidone group, 55.5% in those with no-antifibrotic treatment). The major reason for death was the mortality for IPF related causes (acute exacerbation, suspected acute exacerbation) in both pirfenidone (67.3%) and no-antifibrotic treatment (73.6%, *P* = 0.525) groups (Fig. 2b). The loss of follow-up was recorded in 6.3% of patients on pirfenidone and 15.1% of those on no-antifibrotic treatment (Table 5). Lung transplantation was performed in only 7 (1.8%) patients treated with pirfenidone, whereas the majority of them (4 patients) were transplanted after 24 months after diagnosis.

**Table 5:** Reason for end of follow-up in enrolled IPF patients

	Pirfenidone (*N* = 383)	No-antifibrotics (*n* = 218)	*P*-value (pirfenidone vs no-antifibrotics)
Number of lost patients (%)	141 (36.8%)	161 (73.9%)	< 0.001
Death^a^	101 (26.4%)	121 (55.5%)	< 0.001
< 12 months	37 (9.7%)	32 (14.7%)	< 0.001
12–24 months	39 (10.2%)	23 (10.6%)	
> 24 months	22 (5.7%)	66 (30.3%)	
Lost to follow-up	24 (6.3%)	33 (15.1%)	0.001
< 12 months	11 (2.9%)	16 (7.3%)	0.002
12–24 months	9 (2.3%)	7 (3.2%)	
> 24 months	4 (1.0%)	10 (4.6%)	
Lung transplantation	7 (1.8%)	0 (0.0%)	0.052
< 12 months	1 (0.3%)	0 (0.0%)	0.345
12–24 months	2 (0.5%)	0 (0.0%)	
> 24 months	4 (1.0%)	0 (0.0%)	
Other	9 (2.3%)	7 (3.2%)	0.601
< 12 months	4 (1.0%)	3 (1.4%)	0.885
12–24 months	2 (0.5%)	1 (0.5%)	
> 24 months	3 (0.8%)	3 (1.4%)	

^a^The date of death is missing in 3 patients treated by pirfenidone

## Discussion

This study on a large national real-life IPF cohort showed clinically meaningful reductions in disease progression as assessed by the decline of lung functions in patients with IPF treated 2-yrs with pirfenidone comparing to no-antifibrotics. After 5-yrs, more than half of patients on pirfenidone were alive comparing to one-third on no-antifibrotics. Moreover, our data highlights the importance of the decline in DL_CO_ of ≥10% as a predictor of mortality in pirfenidone treated patients.

Several studies in various populations proved that pirfenidone slows-down the disease progression in terms of declining the lung functions during the first year of administration [11–18]. Nowadays, there is the need for extended follow-up [19]. Since the registration trials of IPF treatments have not been designed to estimate long-term survival, an open-label extension study (RECAP) [29, 30] based on patients from randomised controlled clinical studies CAPACITY and ASCEND [5, 6] has been designed. The RECAP study supports the safety and long-term efficacy of pirfenidone with median survival of 72.2 months, which corresponds to our data where at 5-yrs (60 months) follow-up the median OS has not been reached. Importantly, 55.9% of our patients on pirfenidone were on follow-up for 5-yrs, which is also in line with the RECAP study showing that 49% of patients from the CAPACITY trial were 5-yrs on pirfenidone follow-up [19, 30]. Other recent study estimated the mean life expectancy with pirfenidone to be 8.72 years (95% confidence interval, 7.65–10.15) [8, 31]. Nevertheless, our study brings additional proof-of-effect of the first antifibrotic treatment to the results of randomized controlled trials CAPACITY and ASCEND [5, 6] and their extension study RECAP [29, 30] in real-world settings. These observations further support pirfenidone treatment benefit in patients who continued receiving therapy.

Next, we were wondering about the dynamics of progression during the 2-yr follow-up on pirfenidone in our real-life cohort of IPF patients. Although it is crucially important to assess disease progression in IPF, there is still no consensus how to classify it [32]. The evidence-based guidelines for management of IPF suggests that an absolute decline of FVC (more than 10%) and decline DL_CO_ (more than 15%) is an acceptable method for assessing disease severity and estimating the risk of mortality [21]. However, some studies highlight the clinical utility of marginal declines FVC (more than 5%) and DL_CO_ (more than 10%) [22, 23]. In our patients on pirfenidone, the progression measured by decline in FVC (≥10%) was evident in 5.3% of patients at month 6. Similar observations (5.5%) were reported in a large cohort of 618 patients on pirfenidone in the ASCEND and CAPACITY trials [31, 33]. Also, the number of our patients progressing on pirfenidone in month 12 (10.7%) were in lines with reported data [9, 11, 13–17]. Importantly, almost twice as many patients progress on no-antifibrotic treatment comparing to pirfenidone during 1-yr follow-up.

Our study further focused on the decline of lung functions in IPF patients at 18 and 24 months after pirfenidone administration. Among patients from RECAP cohort, 16.3% experienced FVC decline ≥10% on pirfenidone at week 60 (month 15) [29, 30], compared with 16.6% in our cohort at month 18. Similar data on slow progression on pirfenidone treatment were also reported in current studies on national real-life cohorts of Danish [18] and Greek [12] IPF patients. Our study proved that even 2-years on pirfenidone administration, less than one-quarter of IPF patients progress as assessed by the decline of ≥10% FVC (17.0%) and ≥ 15% DL_CO_ (14.3%). When comparing pirfenidone to no-antifibrotic treatment, twice as many patients progressed on no-antifibrotic treatment in our cohort. The long-term slow-down impairment of lung function on pirfenidone and other antifibrotic treatments has an important issue also in terms of timing for lung transplantation. Prolonged time with no disease progression may increase the chance for lung transplant in IPF patients. Despite the short mean waiting time of 6 months for lung transplantation for patients with interstitial lung diseases (ILD) in the Czech Republic, the number of transplanted patients in our cohort was very low (less than 2%). The main reason for the small number of transplanted patients may be the size of requested donor lungs for IPF patients as well as the current age limit of 65 yrs. for recipients of lung transplant in the Czech Republic.

There is also evidence that pirfenidone therapy is associated with a reduction in the relative risk of mortality compared with placebo over 120 weeks [31]. We were therefore interested whether the decline in lung function on particular treatment may be associated with increased mortality in our patients as shown in previous studies [34, 35]. Indeed, the DL_CO_ (≥10%) declines at 6, 12, 18 and 24 months’ and DL_CO_ (≥15%) decline at 6, 18 and 24 months’ follow-up were associated with increased mortality in our patients on pirfenidone. This finding suggests that in some IPF patients, fibrotic processes ongoing in the lungs are not completely controlled by antifibrotic therapy, which is first seen in changes of DL_CO_. Importantly, the DL_CO_ decline of ≥10% showed higher predictive value for mortality on pirfenidone at all investigated time intervals than FVC decline. This observation is in line with previous studies [35–37], where the DL_CO_ declines were more predictive of mortality risk than FVC ones. In patients with no-antifibrotics, both FVC and DL_CO_ declines were not predictive for mortality. This may be due to the high heterogeneity of lung function impairment in our patients treated with no-antifibrotic drugs, including more than half of patients with severe lung impairment. Our data may also suggest that the decrease in lung function in “severe” IPF is less informative in regards to mortality than in those with “mild” and “moderate” disease. Also the fact that treatment with no-antifibrotics does not lead to functional and symptomatic stabilisation together with higher number of acute exacerbations may contribute to our observation. These results further support the profit of antifibrotic treatments in IPF and need to be validated in future studies. Our study further confirmed the clinical utility of declines in DL_CO_ of more than 10% for the identification of risk patients with poor prognosis on pirfenidone, for whom timely transplantation is required. Therefore, we suggest the DL_CO_ assessment as a part of routine follow-up examinations in IPF patients on pirfenidone. The clinical usefulness the DL_CO_ decline as a mortality predictor deserves future investigation on larger independent cohorts. Moreover, we are aware that this retrospective study requires future testing in a prospectively recruited cohort of patients.

Despite the European Medicines Agency approving and introducing in most European countries antifibrotic treatment of IPF in 2011, many patients with confirmed IPF still do not receive approved antifibrotic treatment as shown by the current study in five European countries (France, Germany, Italy, Spain, and the UK) [38]. According to this study, in 2016 71% of “mild” IPF, 41% of “moderate” and 60% of patients with “severe” IPF did not receive any approved antifibrotic treatment. Also, in our cohort, half of the patients with no-antifibrotic treatment fulfil the Czech criteria for indication of antifibrotic treatment [28]. The majority of those patients were entering the study before introducing the commercially available antifibrotic drug. Moreover, some patients fullfilling the criteria for antifibrotic treatment migtht not receive this drug shortly after its introduction due to the need of establishment of communication between local pneumologists and ILD centres in the Czech Republic, which may only prescribe antifibrotic drugs. Although pirfenidone treatment is mainly recommended for patients with mild to moderate IPF, the recent opinion also suggests the initiation of treatment in patients with severe functional impairment. There is already a growing body of evidence that patients with advanced IPF (FVC < 50% and/or DLco < 35%) profit from pirfenidone leading to functional and symptomatic stabilisation [9–11, 39]. Despite symptomatic stabilisation and slowing down the disease progression, our study showed more deaths in a group of IPF patients with more severe pulmonary function impairment in both pirferidone and no-antifibrotic treatments. Therefore, efforts for early diagnosis of the condition increases as there is evidence about more effective therapeutic efficiency, less complications and lower mortality comparing to severe disease. More studies are needed to include patients with severe functional impairment in antifibrotic treatment indications by the health authorities. Our data further supports the importance of registries in the understanding of this devastating rare disease, disease progression, quality of life and outcomes on the treatment.

## Conclusion

Our data proved that pirfenidone has a 2-yrs lasting effect on the slowing down of lung function decline, as well as on reducing mortality compared to no-antifibrotic treatments in IPF patients from the Czech part of the EMPIRE registry. Our data provide further evidence of the effect of the first antifibrotic treatment in IPF to the results of randomized controlled trials in real-world settings. The 5-yrs OS was markedly higher in patients treated with pirfenidone compared to no-antifibrotic treatment, with more than half of patients on pirfenidone still living comparing to one-third on no-antifibrotics. Finally, the DL_CO_ assessment should be part of routine follow-up investigations in IPF patients on pirfenidone, as its decline was highly associated with mortality and those risk patients are candidates for timely lung transplantation.
